# Sex Differences in Outcomes of Critically Ill Adults with Respiratory Syncytial Virus Pneumonia: A Retrospective Exploratory Cohort Study

**DOI:** 10.3390/idr17060151

**Published:** 2025-12-18

**Authors:** Josef Yayan, Kurt Rasche

**Affiliations:** Department of Internal Medicine, Division of Pulmonary, Allergy and Sleep Medicine, HELIOS Clinic Wuppertal, Witten/Herdecke University, Heusnerstr. 40, 42283 Wuppertal, Germany

**Keywords:** respiratory syncytial virus, pneumonia, intensive care, critical care outcomes, sex differences, mortality, mechanical ventilation, weaning, MIMIC-IV database

## Abstract

**Background:** Respiratory syncytial virus (RSV) pneumonia is an underrecognized cause of critical illness in adults. However, the influence of biological sex on intensive care unit (ICU) outcomes in this population remains unclear. Due to limited case numbers and incomplete covariate data, this study was designed as exploratory and hypothesis-generating. **Methods:** We conducted a retrospective exploratory cohort study using the MIMIC-IV database and identified 105 adult ICU patients with laboratory-confirmed RSV pneumonia. Clinical variables included sex, age, ICU length of stay, use of mechanical ventilation, and weaning status. Exploratory multivariable logistic regression was performed to assess associations with in-hospital mortality and weaning success, acknowledging substantial missingness of comorbidity data, severity scores, and treatment variables. This limited adjustment for confounding and statistical power. **Results:** Overall, in-hospital mortality was 33.3%. Mortality was significantly higher among women than men (51.6% vs. 7.0%; *p* < 0.001), although the absolute number of deaths in men was very small. In adjusted models, female sex (OR 14.6, 95% CI 1.58–135.3, *p* = 0.018), reflecting model instability due to sparse events, as well as longer ICU stay (OR 1.22 per day, *p* = 0.001) were independently associated with higher mortality. Female sex was also associated with lower odds of successful weaning (OR 0.07, 95% CI 0.01–0.63, *p* = 0.018). These effect estimates must be interpreted cautiously due to the very small number of deaths in men and the resulting wide confidence intervals. Age and ventilation duration were not significant predictors. **Conclusions:** In this preliminary ICU cohort, female sex and prolonged ICU stay were linked to higher mortality and lower weaning success in adults with RSV pneumonia. However, given the very small number of events—particularly among male patients—together with the modest sample size, limited covariate availability, and unstable effect estimates, the findings should be viewed as exploratory rather than confirmatory. Larger, well-powered, prospective multicenter studies are needed to validate and further characterize potential sex-related differences in outcomes of RSV-associated critical illness.

## 1. Introduction

Respiratory syncytial virus (RSV) is a leading cause of acute respiratory tract infections worldwide. Although historically regarded as a pediatric pathogen [1,2], RSV is increasingly recognized as a major source of severe respiratory disease in adults—particularly among the elderly and those with chronic comorbidities such as chronic obstructive pulmonary disease (COPD), cardiovascular disease, or immunosuppression [3,4,5]. In these vulnerable groups, RSV pneumonia can progress to life-threatening lower respiratory tract involvement that often requires hospitalization and, in severe cases, admission to the intensive care unit (ICU) [6,7].

Despite the growing burden of RSV in critically ill adults, prognostic factors and outcome determinants in this population remain insufficiently characterized. Most existing evidence stems from pediatric cohorts or mixed viral pneumonia studies [8,9,10,11]. Consequently, the predictors of mortality and recovery in adult ICU patients with RSV pneumonia—particularly factors related to biological sex—are not well understood. This knowledge gap is especially relevant because sex-related differences in disease severity may be influenced by multiple potentially unmeasured clinical factors, including comorbidity burden, baseline frailty, and severity of acute illness, which are incompletely captured in many large databases.

Sex influences immune regulation, hormonal signaling, and gene expression, thereby affecting host responses to viral infections [12,13,14]. Pronounced sex-based differences have been reported in influenza, SARS-CoV-2, and other respiratory infections [15,16], yet their potential role in RSV-associated critical illness has received little systematic investigation. While several biological mechanisms—such as hormonal modulation of antiviral immunity or sex-specific inflammatory responses—have been proposed, these pathways have not been evaluated in adult RSV cohorts and remain largely speculative in the absence of appropriately designed mechanistic studies. Clarifying whether sex modulates ICU outcomes in RSV pneumonia could improve risk stratification and inform individualized management strategies.

To address this knowledge gap, we conducted an exploratory, retrospective cohort study using the publicly available Medical Information Mart for Intensive Care IV (MIMIC-IV) database [17]. We aimed to evaluate whether sex and age were associated with in-hospital mortality and weaning outcomes among adult ICU patients with RSV pneumonia. Because MIMIC-IV contains limited information on comorbidities, standardized severity scores (e.g., SOFA, APACHE II), and treatment variables, and because the number of eligible RSV cases is small, the study was intentionally designed as a hypothesis-generating analysis rather than a definitive assessment of causal relationships. By highlighting possible sex-related patterns in RSV outcomes, this study seeks to provide preliminary evidence to guide future, more comprehensive investigations in larger, prospectively collected multicenter cohorts.

## 2. Material and Methods

### 2.1. Study Design and Data Source

We performed a retrospective, hypothesis-generating cohort analysis using the Medical Information Mart for Intensive Care IV (MIMIC-IV), version 2.0 (Massachusetts Institute of Technology, Cambridge, MA, USA). This publicly available, de-identified database contains comprehensive demographic, clinical, and outcome data from more than 60,000 ICU admissions at Beth Israel Deaconess Medical Center (Boston, MA, USA) between 2008 and 2019 [17]. All analyses were conducted in accordance with the MIMIC data-use agreement. Because the dataset is fully anonymized, institutional review board approval and informed consent were not required.

### 2.2. Patient Identification and Eligibility

Adult patients (≥18 years) were screened for RSV pneumonia using International Classification of Diseases, Tenth Revision (ICD-10) diagnostic codes J12.1 (“Respiratory syncytial virus pneumonia”) and B97.4 (“Respiratory syncytial virus as the cause of diseases classified elsewhere”) combined with documented positive microbiological confirmation of RSV. Positive tests included polymerase chain reaction (PCR) assays and viral antigen detection recorded in respiratory specimens such as nasopharyngeal swabs, tracheal aspirates, or bronchoalveolar lavage fluid.

To minimize the risk of identifying patients in whom RSV was only a secondary diagnosis, cases coded with B97.4 were included only when a concurrent lower respiratory-tract infection was documented in the clinical notes or when RSV-positive respiratory samples were obtained within 48 h prior to or after ICU admission.

Cases with ICU stays shorter than 24 h or with missing mortality or ventilation data were excluded. For individuals with multiple ICU admissions, only the first qualifying admission was analyzed to avoid duplication.

### 2.3. Variables and Outcomes

Baseline variables included age, sex, ICU admission and discharge dates, duration of invasive mechanical ventilation, and total ICU length of stay. The primary outcomes were (1) in-hospital mortality and (2) weaning outcomes. Weaning success was defined as survival following discontinuation of invasive mechanical ventilation, whereas weaning failure denoted death during ventilatory support.

Data on illness severity scores (Sequential Organ Failure Assessment [SOFA], Acute Physiology and Chronic Health Evaluation II [APACHE II]), comorbidities, laboratory biomarkers, viral load, co-infections, and pharmacologic or supportive therapies were frequently incomplete or unavailable in MIMIC-IV and therefore could not be incorporated. To quantify the extent of missingness, we assessed the availability of key clinical variables and found that more than 60–80% of entries were incomplete. This substantial level of missing data precluded reliable covariate adjustment or multiple imputation and represents a major limitation of the analysis, markedly constraining our ability to control for confounding.

### 2.4. Statistical Analysis

Continuous variables were summarized as mean ± standard deviation (SD) or median (interquartile range [IQR]) as appropriate; categorical variables were expressed as counts and percentages. Between-group comparisons employed the Student’s t test or Mann–Whitney U test for continuous variables and the chi-square or Fisher’s exact test for categorical variables.

Univariable logistic regression identified potential predictors of in-hospital mortality and weaning outcomes. Variables demonstrating clinical plausibility or statistical significance (*p* < 0.10) in univariable analyses were entered into multivariable logistic regression models. Adjustment involved simultaneous inclusion of age, ICU length of stay, and ventilation duration in the regression models; no interaction terms were applied due to the limited number of outcome events. Odds ratios (OR) with 95% confidence intervals (CI) were reported. Model fit was evaluated using the Hosmer–Lemeshow goodness-of-fit test and variance inflation factors to assess multicollinearity.

All analyses were performed using R version 4.3.2 (R Foundation for Statistical Computing, Vienna, Austria). Results from preliminary calculations conducted with VassarStats were confirmed in R to ensure robustness. A two-sided *p* value < 0.05 was considered statistically significant.

## 3. Results

### 3.1. Cohort Characteristics

A total of 105 adult ICU patients with confirmed RSV pneumonia met inclusion criteria, comprising 43 men (41.0%) and 62 women (59.0%), with a mean age of 66.6 ± 13.4 years. Female patients were significantly older than male patients (70.1 ± 11.4 vs. 61.7 ± 14.5 years; *p* < 0.001). Baseline demographic and clinical characteristics are summarized in Table 1. Because detailed comorbidity and severity-of-illness data were unavailable for most patients, these characteristics could not be compared between groups.

### 3.2. Sex Differences in Mortality

Overall, in-hospital mortality was 33.3% (35/105). Mortality was markedly higher among women (51.6%, 32/62) compared with men (7.0%, 3/43) (*p* < 0.001). In univariable logistic regression, female sex was associated with greater odds of death (OR 10.7, 95% CI 1.99–57.5, *p* = 0.006) (Table 2). Given the very small number of deaths among men, these estimates should be interpreted cautiously due to limited statistical stability.

### 3.3. Predictors of Mortality

After adjusting for age, ICU length of stay, and duration of ventilation, female sex remained independently associated with increased mortality (OR 14.6, 95% CI 1.58–135.3, *p* = 0.018). Longer ICU stay also predicted mortality (OR 1.22 per day, 95% CI 1.08–1.38, *p* = 0.001). Age and ventilation duration were not significant predictors (Table 3). The wide confidence intervals reflect the small sample size and limited number of events, especially among men, and therefore indicate reduced precision and potential model instability.

### 3.4. Mechanical Ventilation and Mortality

No significant association was found between invasive mechanical ventilation and in-hospital mortality (OR 1.0, 95% CI 0.09–11.42, *p* = 1.0) (Table 4). However, because only a very small number of patients were not mechanically ventilated, the statistical estimate is highly imprecise, and this finding should therefore be interpreted with particular caution.

### 3.5. Age-Related Outcomes

Unexpectedly, patients younger than 65 years had higher mortality compared with those ≥ 65 years (53.8% vs. 13.2%; *p* < 0.001), despite significantly longer ICU stays in the younger group (16.3 ± 12.3 vs. 5.6 ± 6.1 days; *p* < 0.001). Ventilation duration did not differ significantly between age groups (Table 5). Because this age-related pattern contradicts what is typically observed in critical illness, it most likely reflects selection bias and substantial residual confounding rather than a true survival disadvantage among younger patients.

### 3.6. Weaning Outcomes

Among 105 patients, 68 (64.8%) were successfully weaned from invasive ventilation, whereas 37 (35.2%) experienced weaning failure (death during ventilation). Weaning failure was substantially more frequent in women (94.1%) than in men (42.6%) (*p* < 0.001). It was also associated with prolonged ICU stay (22.8 ± 10.4 vs. 5.4 ± 5.4 days; *p* < 0.001) and longer ventilation duration (3.1 ± 3.7 vs. 1.4 ± 2.9 days; *p* = 0.013) (Table 6).

In multivariable analysis, female sex (OR 0.07, 95% CI 0.01–0.63, *p* = 0.018) and longer ICU stay (OR 0.82 per day, 95% CI 0.73–0.92, *p* = 0.001) remained independently associated with lower odds of successful weaning, whereas age and ventilation duration were not significant predictors (Table 7). The wide confidence intervals again underscore the exploratory nature of these findings and highlight the limited statistical precision due to the small number of events.

**Figure 1:**
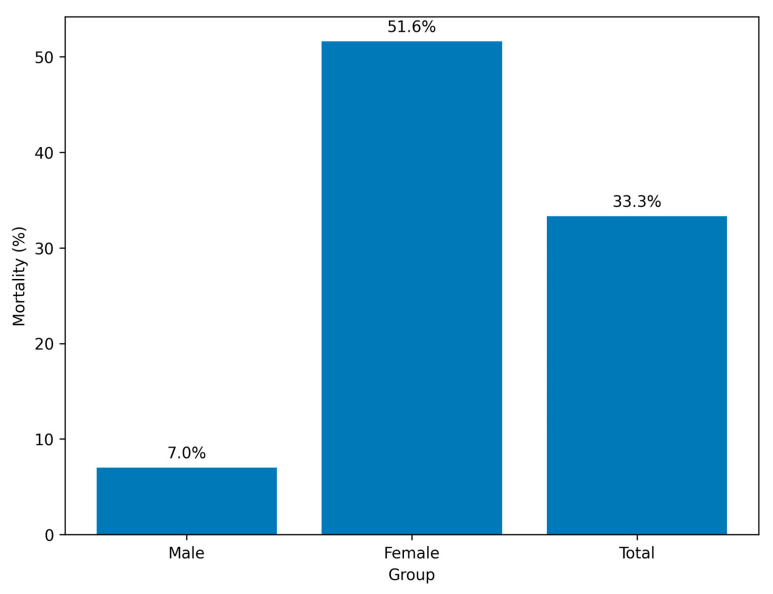
In-hospital mortality among intensive care unit (ICU) patients with respiratory syncytial virus (RSV) pneumonia, stratified by sex. Mortality was 7.0% in men and 51.6% in women, resulting in an overall mortality rate of 33.3%.

**Figure 2:**
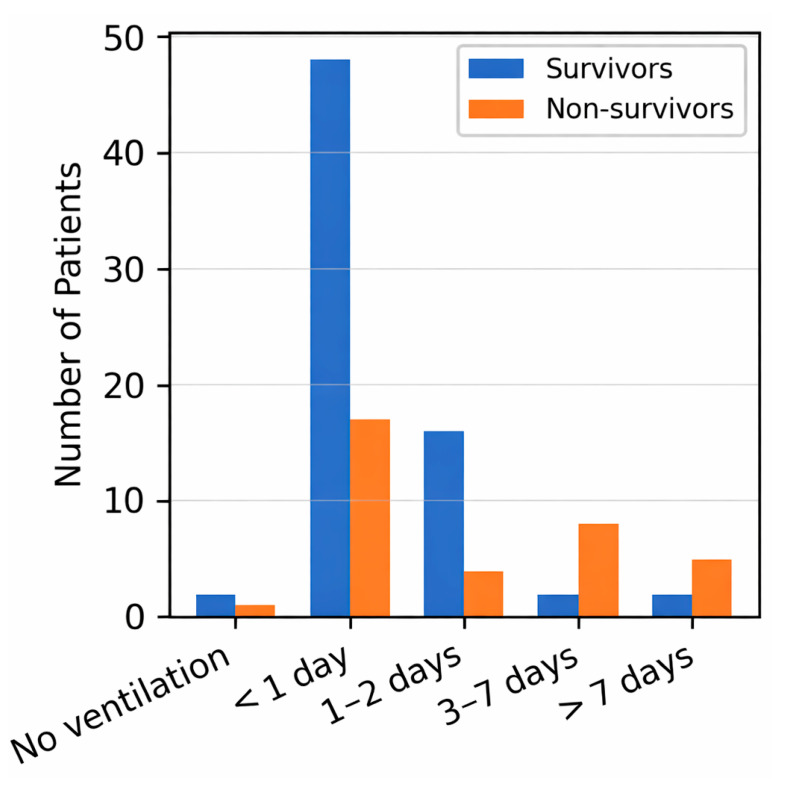
Duration of invasive mechanical ventilation stratified by survival status in intensive care unit (ICU) patients with respiratory syncytial virus (RSV) pneumonia. Blue bars represent survivors, and orange bars represent non-survivors. The x-axis indicates ventilation duration (days), and the y-axis shows the number of patients.

To avoid unnecessary redundancy, Figure 1 (mortality by sex) and Figure 2 (ventilation duration by survival status) are included for visual illustration only, while all numerical data are fully reported in the corresponding tables.

## 4. Discussion

In this retrospective exploratory cohort study of critically ill adults with RSV pneumonia, we observed pronounced sex-related differences in short-term outcomes. Female patients had significantly higher in-hospital mortality and markedly lower rates of successful weaning from invasive ventilation than male patients, despite comparable ventilation durations. These findings raise the possibility that biological sex influences the clinical trajectory of RSV-associated critical illness; however, the observational design and limited covariate availability preclude any causal interpretation, and the results should be viewed as preliminary.

The overall mortality rate of 33.3% exceeds that reported for general hospitalized RSV populations, reflecting the high severity of infection in ICU settings [18]. The observed mortality difference between women (≈52%) and men (≈7%) persisted after adjustment for age, ICU stay, and ventilation duration, but the wide confidence intervals and small number of deaths—particularly among men—indicate substantial statistical fragility. The unusually large effect estimates (e.g., OR 14.6 for female sex) likely reflect sparse events and model instability rather than a robust underlying biological difference. The results should therefore be interpreted as hypothesis-generating rather than confirmatory.

The present study represents one of the first analyses of sex-specific outcomes in adult ICU patients with RSV pneumonia using the MIMIC-IV database, but several limitations restrict interpretability. Key clinical covariates—including comorbidities, illness-severity scores (SOFA, APACHE II), laboratory parameters, co-infections, and therapeutic interventions—were incompletely recorded and thus could not be incorporated. The extensive missingness of these variables introduces considerable residual confounding and may have influenced both the observed sex differences and the associations with mortality and weaning outcomes. The small cohort size further limited the stability of multivariable models, as reflected by the broad confidence intervals, and precluded comprehensive sensitivity analyses. These constraints reduce statistical power and increase the likelihood of biased parameter estimates.

Although biologically plausible mechanisms such as hormonal and immune modulation by sex steroids have been proposed [19,20,21,22,23], our dataset cannot test these pathways. Consequently, any mechanistic interpretation remains speculative and should be explored in appropriately designed translational studies. 

Although clinical practice guidelines exist for the management of hospital-acquired and ventilator-associated pneumonia, they do not specifically address RSV-related critical illness, limiting their applicability in this context [24]. As a result, management strategies for severe RSV infection in adult ICU patients are largely extrapolated from broader pneumonia guidelines rather than supported by virus-specific evidence.

Respiratory syncytial virus infection in elderly and high-risk adults is generally associated with substantial morbidity and mortality; however, progression to critical illness requiring intensive care represents a distinct and more severe disease spectrum [25]. Our findings underscore the importance of focusing specifically on ICU cohorts to better understand outcome determinants in severe adult RSV infection.

Prolonged ICU stay was independently associated with both mortality and weaning failure, likely reflecting cumulative burdens of secondary infection, organ dysfunction, and frailty [26]. Given that ICU length of stay is not an exogenous variable but may itself be influenced by complications or delayed recovery, its association with mortality must be interpreted cautiously to avoid circular inference. The longer stays observed among female patients may partly explain their poorer outcomes, underscoring the interplay between biological, clinical, and care-process factors.

Likewise, the paradoxically higher mortality among younger patients (<65 years) may result from selection or coding bias, differences in case ascertainment, or unmeasured clinical severity rather than true age-related differences in susceptibility [27,28].

The absence of a clear relationship between mechanical ventilation and mortality suggests that extrapulmonary complications, rather than respiratory failure alone, may drive adverse outcomes in this population. Nonetheless, the markedly higher weaning failure among women aligns with prior evidence of sex-based variation in ventilatory mechanics and recovery in other ICU cohorts [29,30], although the wide confidence intervals again limit the certainty of this conclusion.

Taken together, these results highlight sex as a potential, yet incompletely understood, determinant of prognosis in adult RSV pneumonia requiring intensive care. Because of the study’s retrospective design, modest sample size, and incomplete covariate capture, the findings should be regarded as preliminary. Larger, prospective multicenter studies with standardized severity assessment, comprehensive comorbidity profiling, detailed treatment information, and statistically robust modeling techniques are essential to validate these associations and clarify the biological and clinical mechanisms underlying potential sex-specific differences in outcomes in RSV-related critical illness.

## 5. Limitations

This study has several important limitations that should be acknowledged. First, its retrospective, observational design precludes causal inference and carries inherent risks of selection bias, information bias, and residual confounding. Although multivariable regression was applied, the models were constrained by the absence of several critical variables. Standardized severity indices (e.g., SOFA, APACHE II), comorbidity profiles, and key laboratory parameters were not consistently available in the MIMIC-IV dataset and therefore could not be incorporated. More than half of these variables were missing, which limited the ability to adjust for important confounders and likely contributed to the instability of effect estimates—particularly the unusually large odds ratios and wide confidence intervals observed for female sex. This limitation must be considered when interpreting the sex-specific associations reported in this study. Second, the analysis was derived from a single tertiary academic center. While MIMIC-IV provides detailed and high-quality ICU data, the single-center nature limits generalizability to broader or community-based populations. Moreover, the database does not include information on viral load, concomitant infections (e.g., bacterial or influenza co-infections), or therapeutic interventions such as antivirals, corticosteroids, and supportive strategies. These unmeasured variables may have influenced outcomes and could confound the observed associations, particularly if treatment practices differed systematically between subgroups. Third, the relatively small cohort—particularly the low number of deaths among men and the limited number of weaning failures—restricted statistical power and precision, resulting in wide confidence intervals. This is exemplified by the extremely broad confidence intervals in the regression models and the potential for quasi-complete separation, both of which reduce the reliability of effect estimates. As such, the regression models should be viewed as exploratory and hypothesis-generating rather than definitive. Additionally, ICU length of stay—identified as a predictor of both mortality and weaning outcomes—is not an exogenous variable; it may itself be influenced by complications, illness severity, and treatment decisions. This introduces the possibility of circular inference and underscores the need for cautious interpretation. Finally, follow-up was limited to the index hospitalization. Long-term outcomes such as post-discharge recovery, recurrence, and quality of life could not be assessed. Future research should prioritize large, prospective, multicenter studies that include comprehensive severity and comorbidity data, standardized treatment variables, and longitudinal follow-up. Such efforts are essential to confirm or refute the preliminary associations observed here and to clarify the biological and clinical mechanisms underlying sex-related disparities in critically ill adults with RSV pneumonia.

## 6. Conclusions

In this retrospective exploratory analysis of adult ICU patients with RSV pneumonia, female sex and prolonged ICU stay were associated with higher in-hospital mortality and lower likelihood of successful weaning from mechanical ventilation. These associations persisted after adjustment for age and ventilation duration but were derived from a modest sample with limited covariate data and wide confidence intervals, underscoring the need for cautious interpretation. The findings indicate a potential influence of sex on outcomes in RSV-related critical illness; however, the underlying mechanisms remain unclear and may be substantially confounded by unmeasured factors such as comorbidities, baseline severity of illness, and treatment variability. Rather than establishing causality, this study provides preliminary evidence to guide future hypothesis-driven research. Prospective multicenter investigations with larger sample sizes, standardized severity assessments, and more complete clinical and treatment data are essential to validate these observations and to elucidate the biological and clinical pathways that may underlie sex-specific differences in outcomes among critically ill adults with RSV pneumonia.

## Figures and Tables

**Table 1:** Demographic and clinical characteristics of intensive care unit (ICU) patients with respiratory syncytial virus (RSV) pneumonia. Data are presented as number (n), mean ± standard deviation (SD), median [range], and percentage where appropriate. ICU stay duration refers to the total length of ICU admission in days. Mortality indicates in-hospital death during the index admission.

Sex	Number of Patients	Mean Age (Years) ± SD	Median Age [Range]	Number of Deaths	Mortality (%)	Mean ICU Stay (Days) ± SD	Median ICU Stay (Days)
Male	43	61.7 ± 14.5	64.0 [25–81]	3	7.0	6.1 ± 6.5	4.6 [0.5–34.0]
Female	62	70.1 ± 11.4	68.0 [18–91]	32	51.6	14.2 ± 12.3	7.5 [0.7–31.8]
Total	105	66.6 ± 13.4	66.0 [18–91]	35	33.3	10.9 ± 11.0	5.7 [0.5–34.0]

**Table 2:** Univariable logistic regression analysis of demographic and clinical variables associated with in-hospital mortality in intensive care unit (ICU) patients with respiratory syncytial virus (RSV) pneumonia. Odds ratios (OR) are presented with 95% confidence intervals (CI) and *p* values. Age refers to the effect per one-year increase; female sex is compared with male sex; and ICU stay duration reflects the effect per additional day in the ICU.

Variable	Odds Ratio (OR)	95% CI (Lower)	95% CI (Upper)	*p* Value
Constant	0.06	0.00	1.95	0.1133
Age (per year)	0.98	0.93	1.03	0.4294
Female sex (vs. male)	10.7	1.99	57.5	**0.0057**
ICU stay duration (per day)	1.16	1.09	1.24	**<0.0001**

Abbreviations: CI: confidence interval; ICU: intensive care unit; *p* value: probability value. Note: statistically significant *p* values (<0.05) are shown in bold.

**Table 3:** Multivariable logistic regression analysis identifying independent predictors of in-hospital mortality in intensive care unit (ICU) patients with respiratory syncytial virus (RSV) pneumonia. Odds ratios (OR) are presented with 95% confidence intervals (CI) and *p* values. Age and ICU stay duration were analyzed as continuous variables; female sex was compared with male sex; and ventilation duration was analyzed as a continuous predictor. The intercept represents the model constant without direct clinical interpretation.

Variable	Odds Ratio (OR)	95% CI (Lower)	95% CI (Upper)	*p* Value
Constant	0.05	0.00	8.65	0.2553
Age (per year)	0.98	0.90	1.05	0.5441
Female sex (vs. male)	14.6	1.58	135.29	**0.0183**
ICU stay duration (per day)	1.22	1.08	1.38	**0.0012**
Ventilation duration (per day)	0.87	0.66	1.15	0.3237

Abbreviations: CI, confidence interval; ICU, intensive care unit; *p* value, probability value. Note: statistically significant *p* values (<0.05) are shown in bold.

**Table 4:** Logistic regression analysis of the association between invasive mechanical ventilation and in-hospital mortality in intensive care unit (ICU) patients with respiratory syncytial virus (RSV) pneumonia. Mechanical ventilation was defined as any recorded non-zero duration of invasive respiratory support. Results are presented as odds ratio (OR) with 95% confidence interval (CI) and *p* value.

Variable	Odds Ratio (OR)	95% CI (Lower)	95% CI (Upper)	*p* Value
Mechanical ventilation (yes vs. no)	1.0	0.09	11.42	1.0

Abbreviations: OR, odds ratio; CI, confidence interval; *p* value, probability value.

**Table 5:** Comparison of demographic and clinical characteristics between patients aged <65 years and those aged ≥65 years with respiratory syncytial virus (RSV) pneumonia in the intensive care unit (ICU). Data are presented as mean ± standard deviation (SD) for continuous variables and percentages for categorical variables. *p* values were calculated using Student’s t test for continuous variables and chi-square test for categorical variables.

Variable	<65 Years	≥65 Years	*p* Value
Age (years) ± SD	56.5 ± 10.8	76.6 ± 6.3	**<0.0001**
Female sex (%)	57.7	60.4	0.9352
ICU stay (days) ± SD	16.3 ± 12.3	5.6 ± 6.1	**<0.0001**
Ventilation duration (days) ± SD	2.2 ± 3.1	1.8 ± 3.4	0.5203
Mortality (%)	53.8	13.2	**<0.0001**

Abbreviations: SD, standard deviation; ICU, intensive care unit; *p* value, probability value. Note: statistically significant *p* values (<0.05) are shown in bold.

**Table 6:** Comparison of demographic and clinical characteristics between patients with successful and failed weaning from invasive mechanical ventilation in the intensive care unit (ICU) cohort with respiratory syncytial virus (RSV) pneumonia. Weaning success was defined as survival following discontinuation of invasive ventilation, while weaning failure was defined as death during invasive ventilation. Data are presented as mean ± standard deviation (SD) for continuous variables and percentages for categorical variables. *p* values were calculated using Student’s t test or chi-square test, as appropriate.

Variable	Weaning Success (N = 68)	Weaning Failure (N = 37)	*p* Value
Age (years) ± SD	68.1 ± 14.7	65.4 ± 6.8	0.3078
Female sex (%)	42.6	94.1	**<0.0001**
ICU stay (days) ± SD	5.4 ± 5.4	22.8 ± 10.4	**<0.0001**
Ventilation duration (days) ± SD	1.4 ± 2.9	3.1 ± 3.7	**0.0129**

Abbreviations: SD, standard deviation; ICU, intensive care unit; *p* value, probability value. Note: statistically significant *p* values (<0.05) are shown in bold.

**Table 7:** Multivariable logistic regression analysis of independent predictors of successful weaning from invasive mechanical ventilation in intensive care unit (ICU) patients with respiratory syncytial virus (RSV) pneumonia. Odds ratios (OR) are presented with 95% confidence intervals (CI) and *p* values. Age, ICU stay duration, and ventilation duration were analyzed as continuous variables; female sex was compared with male sex. The intercept represents the model constant without direct clinical interpretation.

Variable	Odds Ratio (OR)	95% CI (Lower)	95% CI (Upper)	*p* Value
Constant	19.78	0.12	3385.83	0.2553
Age (per year)	1.02	0.95	1.11	0.5441
Female sex (vs. male)	0.07	0.01	0.63	**0.0183**
ICU stay duration (per day)	0.82	0.73	0.92	**0.0012**
Ventilation duration (per day)	1.15	0.87	1.52	0.3237

Note: statistically significant *p* values (<0.05) are shown in bold.

## Data Availability

The datasets analyzed in this study are publicly available through the MIMIC-IV database (https://mimic.physionet.org) (accessed on 25 September 2025).
